# Xpert MTB/RIF Ultra for detection of *Mycobacterium tuberculosis* and rifampicin resistance: a prospective multicentre diagnostic accuracy study

**DOI:** 10.1016/S1473-3099(17)30691-6

**Published:** 2018-01

**Authors:** Susan E Dorman, Samuel G Schumacher, David Alland, Pamela Nabeta, Derek T Armstrong, Bonnie King, Sandra L Hall, Soumitesh Chakravorty, Daniela M Cirillo, Nestani Tukvadze, Nino Bablishvili, Wendy Stevens, Lesley Scott, Camilla Rodrigues, Mubin I Kazi, Moses Joloba, Lydia Nakiyingi, Mark P Nicol, Yonas Ghebrekristos, Irene Anyango, Wilfred Murithi, Reynaldo Dietze, Renata Lyrio Peres, Alena Skrahina, Vera Auchynka, Kamal Kishore Chopra, Mahmud Hanif, Xin Liu, Xing Yuan, Catharina C Boehme, Jerrold J Ellner, Claudia M Denkinger, study team, Susan E Dorman, Samuel G Schumacher, David Alland, Pamela Nabeta, Derek T Armstrong, Bonnie King, Sandra L Hall, Soumitesh Chakravorty, Daniela M Cirillo, Nestani Tukvadze, Nino Bablishvili, Wendy Stevens, Lesley Scott, Camilla Rodrigues, Mubin I Kazi, Moses Joloba, Lydia Nakiyingi, Mark P Nicol, Yonas Ghebrekristos, Irene Anyango, Wilfred Murithi, Reynaldo Dietze, Renata Lyrio Peres, Alena Skrahina, Vera Auchynka, Kamal Kishore Chopra, Mahmud Hanif, Xin Liu, Xing Yuan, Catharina C Boehme, Jerrold J Ellner, Claudia M Denkinger, Yukari C Manabe, David Hom, Rusudan Aspindzelashvili, Anura David, Utkarsha Surve, Louis Henry Kamulegeya, Sheila Nabweyambo, Shireen Surtie, Nchimunya Hapeela, Kevin P Cain, Janet Agaya, Kimberly D McCarthy, Patricia Marques-Rodrigues, Luiz Guilherme Schmidt Castellani, Pedro Sousa Almeida, Paola Poloni Lobo de Aguiar, Varvara Solodovnikova, Xianglin Ruan, Lili Liang, Guolong Zhang, Hong Zhu, Yingda Xie

**Affiliations:** a Johns Hopkins University School of Medicine, Baltimore, MD, USA; b FIND, Geneva, Switzerland; c Division of Infectious Diseases, Rutgers-New Jersey Medical School, Newark, NJ, USA; d Boston Medical Center and Boston University School of Medicine, Boston, MA, USA; e IRCCS San Raffaele Scientific Institute, Milan, Italy; f National Center for Tuberculosis and Lung Diseases, Tbilisi, Georgia; g Department of Molecular Medicine and Haematology, Faculty of Health Science, School of Pathology and the National Priority Program of the National Health Laboratory Service, Johannesburg, South Africa; h PD Hinduja Hospital and Medical Research Centre, Mumbai, India; i Mycobacteriology Laboratory, Department of Microbiology, School of Biomedical Sciences, Makerere University, Kampala, Uganda; j Infectious Disease Institute, Makerere University, Kampala, Uganda; k Division of Medical Microbiology and Institute for Infectious Diseases and Molecular Medicine, University of Cape Town, Cape Town, South Africa; l National Health Laboratory Service, Groote Schuur Hospital, Cape Town, South Africa; m Kenya Medical Research Institute, Center for Global Health Research, Kisumu, Kenya; n Universidade Federal do Espirito Santo, Vitoria, Brazil; o National Reference Laboratory, Republican Scientific and Practical Centre for Pulmonology and Tuberculosis, Minsk, Belarus; p State TB Training & Demonstration Centre, New Delhi, India; q Henan Provincial Chest Hospital, Zhengzhou, Henan Province, China

## Abstract

**Background:**

The Xpert MTB/RIF assay is an automated molecular test that has improved the detection of tuberculosis and rifampicin resistance, but its sensitivity is inadequate in patients with paucibacillary disease or HIV. Xpert MTB/RIF Ultra (Xpert Ultra) was developed to overcome this limitation. We compared the diagnostic performance of Xpert Ultra with that of Xpert for detection of tuberculosis and rifampicin resistance.

**Methods:**

In this prospective, multicentre, diagnostic accuracy study, we recruited adults with pulmonary tuberculosis symptoms presenting at primary health-care centres and hospitals in eight countries (South Africa, Uganda, Kenya, India, China, Georgia, Belarus, and Brazil). Participants were allocated to the case detection group if no drugs had been taken for tuberculosis in the past 6 months or to the multidrug-resistance risk group if drugs for tuberculosis had been taken in the past 6 months, but drug resistance was suspected. Demographic information, medical history, chest imaging results, and HIV test results were recorded at enrolment, and each participant gave at least three sputum specimen on 2 separate days. Xpert and Xpert Ultra diagnostic performance in the same sputum specimen was compared with culture tests and drug susceptibility testing as reference standards. The primary objectives were to estimate and compare the sensitivity of Xpert Ultra test with that of Xpert for detection of smear-negative tuberculosis and rifampicin resistance and to estimate and compare Xpert Ultra and Xpert specificities for detection of rifampicin resistance. Study participants in the case detection group were included in all analyses, whereas participants in the multidrug-resistance risk group were only included in analyses of rifampicin-resistance detection.

**Findings:**

Between Feb 18, and Dec 24, 2016, we enrolled 2368 participants for sputum sampling. 248 participants were excluded from the analysis, and 1753 participants were distributed to the case detection group (n=1439) and the multidrug-resistance risk group (n=314). Sensitivities of Xpert Ultra and Xpert were 63% and 46%, respectively, for the 137 participants with smear-negative and culture-positive sputum (difference of 17%, 95% CI 10 to 24); 90% and 77%, respectively, for the 115 HIV-positive participants with culture-positive sputum (13%, 6·4 to 21); and 88% and 83%, respectively, across all 462 participants with culture-positive sputum (5·4%, 3·3 to 8·0). Specificities of Xpert Ultra and Xpert for case detection were 96% and 98% (−2·7%, −3·9 to −1·7) overall, and 93% and 98% for patients with a history of tuberculosis. Xpert Ultra and Xpert performed similarly in detecting rifampicin resistance.

**Interpretation:**

For tuberculosis case detection, sensitivity of Xpert Ultra was superior to that of Xpert in patients with paucibacillary disease and in patients with HIV. However, this increase in sensitivity came at the expense of a decrease in specificity.

**Funding:**

Government of Netherlands, Government of Australia, Bill & Melinda Gates Foundation, Government of the UK, and the National Institute of Allergy and Infectious Diseases.

## Introduction

An estimated 10·4 million new tuberculosis cases occurred in 2015, but only 6·1 million (59%) were diagnosed.^1^ That same year, an estimated 580 000 rifampicin-resistant cases occurred, but only 125 000 (20%) were identified.^1^ These diagnostic gaps are caused mostly by the lack of highly sensitive, rapid, accessible diagnostics.^2^ WHO recommended the Xpert MTB/RIF assay (Cepheid, Sunnyvale, CA, USA), an automated, integrated, cartridge-based molecular assay, as the initial test for tuberculosis to increase case detection and improve identification of rifampicin resistance directly from sputum.^3^, ^4^, ^5^ Xpert is used in tuberculosis programmes in more than 120 countries.^6^ However, Xpert's sensitivity for tuberculosis detection is inadequate when few bacilli are present in a clinical specimen. This limits the usefulness of Xpert in patients with sputum smear-negative or extrapulmonary tuberculosis. This is particularly relevant for people with HIV and for children, in whom tuberculosis is often difficult to diagnose and morbidity can be high.^7^, ^8^, ^9^ One possible consequence of imperfect test sensitivity is lack of confidence in a negative test result, leading to empiric treatment and possibly overtreatment that might undermine clinical effect.^10^, ^11^ For detection of rifampicin resistance, Xpert can give a false-positive result for strains that carry phenotypically silent mutations or if the bacillary burden is very low, although this is rare.^12^, ^13^

**Research in context**

**Evidence before this study**

In 2010, WHO endorsed the Xpert MTB/RIF assay for initial diagnostic testing of individuals suspected of multidrug-resistant tuberculosis or HIV-associated tuberculosis. In 2014, WHO expanded this recommendation for use in all patients. The diagnostic accuracy of Xpert for pulmonary tuberculosis and rifampicin resistance has been assessed in Cochrane systematic reviews. The most recent update included studies described in any language until Feb 7, 2013. In 27 studies with nearly 10 000 participants, the pooled sensitivities of Xpert for pulmonary tuberculosis were 98% in those who were positive by sputum smear microscopy but only 67% in those who were negative by sputum smear microscopy. Pooled sensitivity was 79% in HIV-positive patients independent of sputum smear status, and pooled specificity was 99%. Performance characteristics for rifampicin resistance were 95% sensitivity and 98% specificity. The suboptimal detection of rifampicin resistance by Xpert in mixed populations containing rifampicin-resistant plus rifampicin-susceptible bacilli and some silent mutations and the consequent false determinations of rifampicin resistance have been confirmed in subsequent reports.

The Xpert MTB/RIF Ultra (Xpert Ultra) assay was developed to overcome the limited sensitivity of Xpert in the detection of pulmonary tuberculosis and limited accuracy of rifampicin resistance detection. We searched PubMed with the term “Xpert MTB/RIF Ultra” for articles in any language published until Oct 18, 2017. Other than two commentaries, we found two primary research articles describing the limit of detection and the performance of Xpert Ultra for detection of *Mycobacterium tuberculosis* in cerebrospinal fluid. Findings from analytical laboratory studies showed that Xpert Ultra had a lower limit of bacillary detection and was more accurate for detection of rifampicin resistance than Xpert. Xpert Ultra was also found to have higher sensitivity than Xpert and culture in paucibacillary specimens of cerebrospinal fluid.

**Added value of this study**

This is the first prospective study on the accuracy of Xpert Ultra for pulmonary tuberculosis. We did this study in eight countries with high burdens of tuberculosis or drug-resistant tuberculosis, and we applied a rigorous reference standard to assure generalisability of the data to the tuberculosis epidemic worldwide. Our findings suggest that Xpert Ultra is substantially more sensitive than Xpert for detection of pulmonary tuberculosis, especially for paucibacillary specimens (ie, smear-negative specimens and specimens from HIV-positive individuals in whom available tests work least well). However, the increased sensitivity of Xpert Ultra came at the expense of a loss of specificity. For detection of rifampicin resistance, Xpert Ultra and Xpert performed comparably.

**Implications of all the available evidence**

The improved sensitivity of the Xpert Ultra assay relative to the Xpert assay should permit more evidence-based treatment decisions and at earlier stages of disease, even in people with HIV who can have high morbidity and mortality from tuberculosis despite relatively low bacillary burdens in sputum. On the basis of these findings, WHO has concluded that Xpert Ultra can be used as an alternative to Xpert for initial testing in adults with signs or symptoms of tuberculosis. Further research in different epidemiological settings and patient populations is needed to clarify the implications of the trade-off between increased sensitivity and decreased specificity and to determine the biological basis for Xpert Ultra-positive and culture-negative results.

The Xpert MTB/RIF Ultra assay (Xpert Ultra) was developed to overcome the limitations of the Xpert assay. To improve assay sensitivity in the detection of *Mycobacterium tuberculosis* complex, Xpert Ultra incorporates two different multicopy amplification targets (IS6110 and IS1081) and uses improved assay chemistry and cartridge design.^14^ These revisions resulted in an approximately 1–log improvement in the lower limit of detection compared with Xpert.^14^ Analytical laboratory data also demonstrated improved differentiation of certain silent mutations, improved detection of rifampicin resistance in mixed infections, and avoidance of false-positive results for detection of rifampicin resistance in paucibacillary specimens.^14^

We compared the diagnostic accuracy of Xpert Ultra with that of Xpert for the detection of pulmonary tuberculosis and rifampicin resistance in a multicentre study in geographically diverse settings, representative of the intended target population for the assay.

## Methods

### Study design and participants

The primary objectives of this initial clinical diagnostic accuracy study were to estimate and compare the sensitivity of a single Xpert Ultra test with that of a single Xpert test of the same raw sputum specimen for detection of smear-negative tuberculosis and rifampicin resistance, and to estimate and compare Xpert Ultra and Xpert specificities for detection of rifampicin resistance. We hypothesised that the sensitivity of a single Xpert Ultra test for detection of smear-negative tuberculosis was non-inferior to that of a single Xpert, and that the sensitivity and the specificity of Xpert Ultra for rifampicin resistance detection were non-inferior to those of Xpert. The study was done at ten reference laboratories in eight countries (South Africa, Uganda, Kenya, India, China, Georgia, Belarus, and Brazil). Eligible study participants were adults presenting at primary health-care centres and hospitals with pulmonary tuberculosis symptoms and who were willing to provide up to four sputum specimens at study enrolment. Participants were recruited prospectively into one of two groups: the case detection group or the multidrug-resistance risk group. Participation in the case detection group required willingness to attend study follow-up visits 42–70 days after enrolment and that no tuberculosis drugs had been taken in the past 6 months. Participants assigned to the multidrug-resistance risk group were at high risk of drug resistance on the basis of one or more of the following criteria: (1) microbiologically confirmed pulmonary tuberculosis with documented rifampicin resistance and tuberculosis treatment received for 31 days or less; (2) known pulmonary tuberculosis with suspected treatment failure; and (3) history of drug-resistant tuberculosis and off tuberculosis treatment for at least 3 months. The study protocol (appendix) was reviewed and approved by ethics committees at study sites and supervising organisations. Written informed consent was obtained from all study participants. Study participation did not affect the standard of care.

### Procedures

Demographic information, medical history, chest imaging results, and HIV test results (at sites where part of routine care) were recorded at enrolment. Participants were asked to provide minimum three sputum specimens on two separate days. Xpert and Xpert Ultra assays, smear microscopy, culture testing, and phenotypic drug susceptibility tests for rifampicin were done on site. Study-specified laboratory quality assurance included the use of external controls (positive and negative) and swab testing of the specimen processing area and of GeneXpert instrument surfaces.

Xpert and Xpert Ultra assays were done by adding sample reagent to the first collected sputum specimen in a 2:1 dilution, and 2·0 mL of the resulting mixture was added to one Xpert and one Xpert Ultra cartridge. Samples were analysed using standard four-module GeneXpert instruments with automated readouts for *M tuberculosis* detection (invalid [no internal assay control detected]; not detected; or detected [with semiquantitation]) and rifampicin resistance (detected, not detected, or indeterminate). The semiquantitative scale for Xpert Ultra results was trace, very low, low, medium, or high. The semiquantitative scale for Xpert results was very low, low, medium, or high.

For reference standard testing, the second and third sputum specimens were first digested with N-acetyl-L-cysteine and sodium hydroxide and concentrated using standard methods.^15^ Smear microscopy was done using Ziehl-Neelsen (Belarus site) or auramine-rhodamine staining (all other sites). 0·5 mL of the resuspended pellet was inoculated into liquid culture using mycobacteria growth indicator tube (MGIT) with a BACTEC 960 instrument (BD Microbiology Systems, Sparks, MD, USA), and 0·2 mL was inoculated on Löwenstein-Jensen medium. Cultures positive for growth of acid-fast bacilli underwent confirmation of *M tuberculosis* complex by MPT64/MPB64 antigen detection^15^ or line probe assays. Phenotypic drug susceptibility testing was done from the first positive *M tuberculosis* culture using the BACTEC MGIT 960 system and a rifampicin critical concentration of 1·0 μg/mL.^15^ Genetic drug susceptibility testing by Sanger DNA sequencing or pyrosequencing of the 81-bp *rpoB* core region was done for cultured isolates from all participants with discordant results between phenotypic drug susceptibility and Xpert Ultra readouts and for a subset of participants with concordant results. Next-generation sequencing or pyrosequencing of IS*6110*, IS*1081*, and *rpoB* from the Xpert Ultra cartridge amplicon was done on specimens for which Xpert Ultra results were positive, but no culture was positive (appendix p 2).

**Figure 1:**
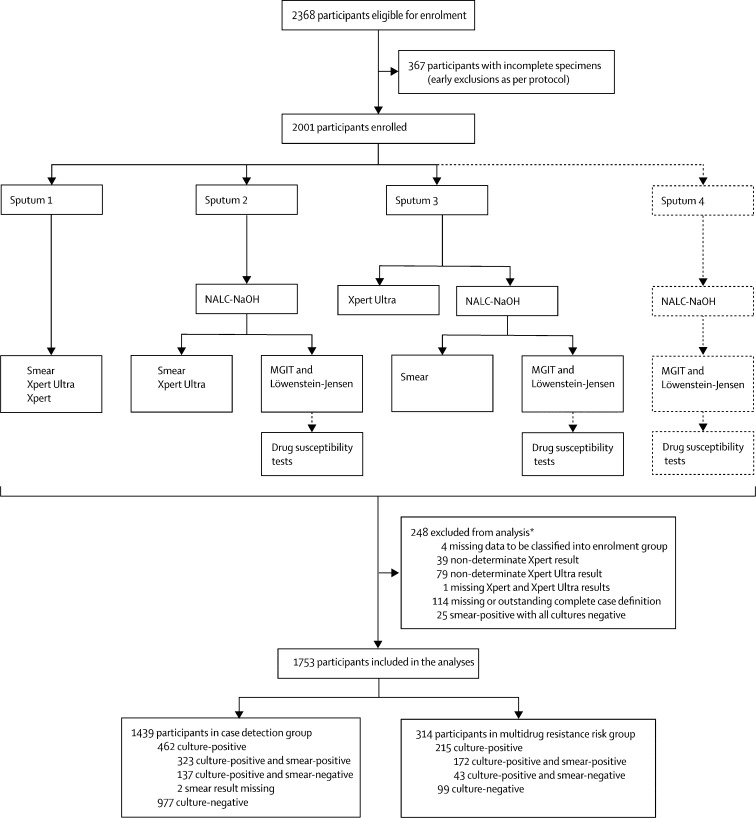
Specimen laboratory testing, participant flow, and exclusions from analysis eligibility Eligible participants were asked to provide four sputum specimens (sputum 1–4) on 2 separate days. Xpert MTB/RIF Ultra assay (Xpert Ultra) on the first of sputum specimen was the index test, and Xpert MTB/RIF assay on the first sputum specimen was the comparator test. When possible, a fourth sputum specimen was obtained for additional solid and liquid cultures in cases with Xpert and Xpert Ultra discrepant results on sputum specimen 1. Sputum 4 results were only used for secondary analyses. NALC-NaOH=N-acetyl-L-cysteine and sodium hydroxide. MGIT=mycobacteria growth indicator tube. *Some reasons for exclusion overlap.

Case definitions for the primary analyses were based on four culture results from sputum specimens two and three (figure 1). A culture-positive tuberculosis case was defined as a participant with at least one culture positive for *M tuberculosis*. Culture-positive cases were considered smear-positive if they had at least one positive smear (inclusive of scanty positive smears). A culture-negative participant had no culture positive for *M tuberculosis* and at least two cultures negative for *M tuberculosis*.

Staff doing Xpert or Xpert Ultra assays were blinded to results of other study tests through use of specimen codes and through staffing assignments. Data were captured through dedicated data-entry systems that were password-protected.

### Statistical analysis

Sample size was calculated by Monte-Carlo Simulation (appendix p 3). Sensitivity was defined as the proportion of patients testing positive with the reference standard who tested positive by the index test (Xpert Ultra) or comparator test (Xpert). Specificity was the proportion of patients testing negative with the reference standard who tested negative by the index test or comparator test. The primary analysis was based on results from initial testing of the first sputum specimen with Xpert and Xpert Ultra. Participants in the case detection group were included in all analyses, whereas participants in the multidrug-resistance risk group were only included in analyses of rifampicin-resistance detection. Patients were excluded from the analysis if culture contamination did not allow application of the case definition or if results of Xpert or Xpert Ultra were indeterminate or missing on initial testing. The proportion testing indeterminate is reported separately.

Results for simple proportions are presented with Clopper-Pearson 95% CI. The 95% CI around differences in proportions (for paired specimens in non-inferiority analyses) was computed using Tango's score method.^16^, ^17^ A non-inferiority endpoint, rather than a superiority endpoint, was selected for this initial clinical diagnostic accuracy study of Xpert Ultra because a superiority endpoint would have required a prohibitively large sample size of enrolled participants with smear-negative pulmonary tuberculosis. We reasoned that assurance from a diagnostic accuracy study that Xpert Ultra was at least as good as Xpert would be useful to clinicians and policy makers and provide rationale for a larger study to assess superiority. Superiority is demonstrated if it can be shown that sensitivity of Xpert Ultra is superior to Xpert beyond what could occur by chance alone. To assess non-inferiority, the lower limit of the CI of the difference in sensitivity (∆) was compared with the predefined non-inferiority margin; non-inferiority is achieved if the lower limit of the CI of ∆ is no lower than the non-inferiority margin. Non-inferiority margins for comparison between Xpert Ultra and Xpert were set at −7% for sensitivity to detect smear-negative tuberculosis, and at −3% for sensitivity and specificity to detect rifampicin resistance (appendix, p 3). A margin was not predefined for specificity of tuberculosis detection. We used Stata version 12 and R version 3.2.4 for statistical analyses.

### Role of the funding source

The funders of the study had no role in study design, data collection, data analysis, data interpretation, or writing of the report. The corresponding author had full access to all the data in the study and had final responsibility for the decision to submit for publication.

## Results

Between Feb 18, and Dec 24, 2016, we enrolled 2368 participants in the study (figure 1). 1753 participants met inclusion criteria and were included in the analyses. Of the 1439 participants in the case detection group, 462 (32%) participants had culture-positive sputum and 137 (30%) participants had smear-negative sputum. Of the 1753 participants in the case detection and multidrug-resistance risk groups, 684 were culture-positive and 213 (31%) of these were rifampicin-resistant on the basis of phenotypic drug susceptibility testing (table 1).

**Table 1:** Demographic and clinical characteristics, enrolment group, and distribution in diagnostic categories of the study participants

	**Minsk, Belarus (N=121)**	**Vitoria, Brazil (N=128)**	**Cape Town, South Africa (N=152)**	**Zheng-zhou, China (N=101)**	**Tbilisi, Georgia (N=372)**	**Johannesburg, South Africa (N=234)**	**Nairobi, Kenya (N=135)**	**Mumbai, India (N=213)**	**New Delhi, India (N=116)**	**Kampala, Uganda (N=181)**	**All participants (N=1753)**
**Demographic or clinical characteristics**											
Age, years	42 (28–56)	50 (37–59)	41 (34–49)	47 (34–57)	45 (33–57)	34 (30–43)	33 (26–44)	31 (23–45)	30 (21–45)	30 (26–39)	38 (28–50)
Female sex	50/121 (41%)	47/128 (37%)	89/152 (59%)	25/101 (25%)	105/372 (28%)	87/234 (37%)	66/135 (49%)	110/213 (52%)	50/116 (43%)	65/181 (36%)	694/1753 (40%)
HIV infection	7/8 (≤4%*)	7/128 (5%)	87/152 (57%)	0/101	7/13 (≤4·0%*)	157/214 (73%†)	78/135 (58%)	8/10 (≤4%*)	7/54 (≤4%*)	83/181 (46%)	441/996 (25%*)
History of tuberculosis‡	5/48 (10%)	10/128 (8%)	59/150 (39%)	1/133 (3%)	95/348 (27%)	55/234 (24%)	20/135 (15%)	7/64 (11%)§	28/115 (24%)	15/181 (8%)	295/1436 (21%)§
**Enrolment group**¶											
Case detection group	48/121 (40%)	128/128 (100%)	150/152 (99%)	33/101 (33%)	348/372 (94%)	234/234 (100%)	135/135 (100%)	67/213 (31%)	115/116 (99%)	181/181 (100%)	1439/1753 (82%)
Multidrug-resistance risk group	73/121 (60%)	0/128	2/152 (1%)	68/101 (67%)	24/372 (6%)	0/234	0/135	146/213 (69%)	1/116 (1%)	0/181	314/1753 (18%)
**Distribution in diagnostic categories**											
Culture-positive sputum‡	25/48 (52%)	34/128 (27%)	27/150 (18%)	26/33 (79%)	95/348 (27%)	74/234 (32%)	28/135 (21%)	44/67 (66%)	43/115 (37%)	67/181 (37%)	462/1439 (32%)
Proportion of participants with culture-positive sputum that was smear-negative‡	14/25 (56%)	5/34 (15%)	14/27 (52%)	4/26 (15%)	39/95 (41%)	18/74 (24%)	6/28 (21%)	14/44 (32%)	8/41 (20%)‖	16/6 (24%)	137/460 (30%)‖
Proportion of participants with culture-positive sputum that was rifampicin resistant**	30/51 (59%)	1/35 (3%)	2/27 (7%)	46/89 (52%)	28/100 (29%)	2/67 (3%)	0/26	92/168 (55%)	11/43 (26%)	0/78	213/684 (31%)

Data are median (IQR) or n/N (%).

* For sites where HIV infection status was unknown for more than 50% of study participants, we show country-level HIV prevalence among tuberculosis cases.

† HIV-infection status was unknown for 20 individuals; data are percentage of patients with known HIV status.

‡ Numbers shown for study participants in the case detection group.

§ Data were missing for three patients at the Mumbai site.

¶ At each site, study participants were enrolled in one of two possible (mutually exclusive) enrolment groups: the case detection group (based on suspicion of tuberculosis) or the multidrug-resistance risk group (based on suspicion of multidrug-resistant tuberculosis).

‖ Smear results were missing for two participants.

** Calculated as percentage of the total number of culture-positive study participants in the case detection group and the multidrug-resistance risk group with available phenotypic drug-susceptibility test results.

Results of the comparison between Xpert and Xpert Ultra sensitivity and specificity are shown in table 2 (appendix p 4). The increase in sensitivity of Xpert Ultra relative to Xpert was larger than the remaining sensitivity gap between Xpert Ultra and a single liquid culture (appendix p 5). Xpert Ultra and Xpert sensitivities using alternative tuberculosis case definitions, as used in previous studies,^3^, ^4^ are shown in the appendix (p 6).

**Table 2:** Comparative accuracy for detection of tuberculosis and rifampicin resistance

	Tuberculosis detection*					**Detection of rifampicin resistance**†	
	Sensitivity: all culture-positive (95% CI; n/N)	Sensitivity: smear-negative (95% CI; n/N)	Sensitivity: HIV-negative (95% CI; n/N)‡	Sensitivity: HIV-positive (95% CI; n/N)‡	Specificity (95% CI; n/N)	Sensitivity (95% CI; n/N)	Specificity (95% CI; n/N)
Xpert	83%(79 to 86; 383/462)	46%(37 to 55; 63/137)§	90%(84 to 94; 143/159)	77%(68 to 84; 88/155)	98%(97 to 99; 960/977)	95%(91 to 98; 167/175)	98%(96 to 99; 369/376)
Xpert Ultra	88%(85 to 91; 408/462)	63%(54 to 71; 86/137)§	91%(86 to 95; 145/159)	90% (83 to 95; 103/115)	96%(94 to 97; 934/977)	95%(91 to 98; 166/175)	98%(97 to 99; 370/376)
Difference (Xpert Ultra minus Xpert)	5·4% (3·3 to 8·0; 25/162)	17% (10 to 24; 23/137)	1·3% (−1·8 to 4·9; 2/159)	13% (6·4 to 21; 15/115)	−2·7% (−3·9 to −1·7; 36/977)	−0·6% (−3·2 to 1·6; 1/175)	0·3% (−0·7 to 1·5; 1/376)
Non-inferiority margin	Not predefined	−7%	Not predefined	Not predefined	Not predefined	−3%	−3%

Results are based on initial testing of the first sample with Xpert MTB/RIF and Xpert MTB/RIF Ultra (Xpert Ultra) assays. Uninterpretable results (contaminated cultures or non-determinate Xpert or Ultra results) were excluded from the analysis. Culture contamination averaged 4·3–7·8%, depending on sample and culture type. Non-determinate results (invalid, error, no result) are reported in the main text. Sensitivities of Xpert and Xpert Ultra for detection of smear-positive tuberculosis (n=323) were 99% (95% CI 97–100) and 99% (97–100).

* Accuracy for tuberculosis detection was estimated in study participants in the case detection group. Patients with unknown HIV-infection status are excluded from analyses stratified by HIV status but included in all other analyses.

† Accuracy for detection of rifampicin resistance was estimated in all study participants with available drug susceptibility test results and valid rifampicin resistance results for both Xpert and Xpert Ultra.

‡ Data on HIV-infection status were not available for 188 culture-positive and 336 culture-negative study participants. Sensitivity of Xpert and Xpert Ultra in study participants with missing HIV status was 81% and 85%, respectively. Note that the estimate for pooled sensitivity of Xpert Ultra irrespective of HIV status does not fall between the estimates for HIV-infected and HIV-uninfected individuals.

§ Accuracy estimates are based on the reference standard as defined in the Methods section (using four cultures to define tuberculosis); using a less stringent reference standard with only one liquid and one solid culture (both from sputum sample 2), which is similar to the reference standard used in 21 of 22 studies included in the most recent Cochrane systematic review of the Xpert assay,^4^ resulted in Xpert sensitivity for smear-negative tuberculosis of 73% (Cochrane review pooled estimate 67%) and Xpert Ultra sensitivity of 84% (appendix p 5).

684 participants had culture-positive sputum and had phenotypic drug susceptibility test results. Xpert Ultra provided interpretable rifampicin drug susceptibility test results for 588 participants (86%), whereas Xpert provided results for 580 participants (85%; appendix p 14). The comparison of sensitivity and specificity between Xpert and Xpert Ultra in the detection of rifampicin resistance is shown in table 2. Incorporating sequencing data for specimens that tested positive for rifampicin resistance by Xpert or Xpert Ultra but rifampicin-susceptible by phenotypic drug susceptibility testing gave specificity estimates of more than 99% for Xpert Ultra and Xpert, which were largely attributable to detection of mutations CTG533CCG, CAC526AAC, and CTG511CCG by Xpert Ultra and Xpert (appendix p 15).

**Figure 2:**
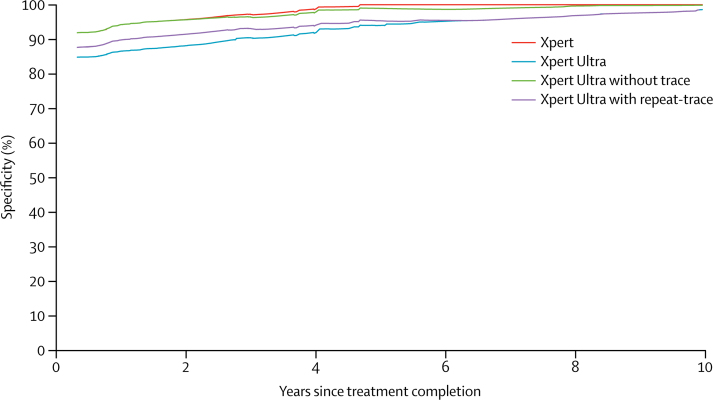
Specificity estimates of Xpert MTB/RIF and Xpert MTB/RIF Ultra (Xpert Ultra) for tuberculosis case detection in patients with a tuberculosis treatment history and for different approaches to handling an initial Xpert Ultra trace-positive result The curves show specificity in participants with tuberculosis history as a function of the time since completion of treatment for the previous tuberculosis episode within 10 years of enrolment (50 cases with treatment more than 10 years earlier were omitted; recoding these 50 cases to be at 10 years did not lead to any noticeable changes in the findings). The results of the Xpert Ultra conditional trace results approach are not shown but would have been directly below the curve for the Ultra without trace. Curves were created using running-line least squares (mean) smoothers with a bandwidth of 0·8.^18^

Results of a predefined subanalysis to compare Xpert Ultra and Xpert specificities in participants in the case detection group with a history of tuberculosis treatment versus no history of tuberculosis treatment are shown in table 3 (appendix p 7). In participants with a history of prior tuberculosis treatment, the reduction in Xpert Ultra specificity was greatest for those who had recently completed their tuberculosis treatment (figure 2; appendix p 8) and only approached the specificity of those without a history of tuberculosis if the previous tuberculosis treatment was at least 7 years before enrolment.

**Table 3:** Test sensitivity and specificity depending on tuberculosis history and different approaches to the interpretation of semiquantitative trace-positive results for *Mycobacterium tuberculosis* detection by Xpert MTB/RIF Ultra (Xpert Ultra)

	**Sensitivity**		**Specificity**		
	All culture-positive (95% CI; n/N)	Smear-negative, culture-positive (95% CI; n/N)	All culture-negative (95% CI; n/N)	No history of tuberculosis (95% CI; n/N)	Any history of tuberculosis (95% CI; n/N)
Xpert	83%(79–86; 383/462)	46%(37–55; 63/137)	98% (97–99; 960/977)	98%(97–99; 715/727)	98%(95–99; 244/249)
Xpert Ultra	88%(85–91; 408/462)	63%(54–71; 86/137)	96% (94–97; 934/977)	96%(95–98; 701/727)	93%(89–96; 232/249)
Xpert Ultra, no trace*	86%(82–89; 395/462)	54%(45–63; 74/137)	98% (96–98; 953/977)	98%(96–99; 709/727)	98%(95–99; 243/249)
Xpert Ultra, conditional trace†	88%(85–91; 406/462)	61%(53–70; 84/137)	97% (95–98; 945/977)	96%(95–98; 701/727)	98%(95–99; 243/249)
Xpert Ultra, trace-repeat‡	87%(84–90; 404/462)	61%(52–69; 83/137)	97% (95–98; 944/977)	97%(96–98; 707/727)	95%(91–97; 236/249)

Sensitivity varied little by history of tuberculosis and did not vary systematically. Data on tuberculosis history were not available for one patient.

* Study participants testing tuberculosis-positive based on a trace-positive Xpert Ultra result (n=32) were reclassified as tuberculosis-negative.

† Study participants testing tuberculosis-positive based on a trace-positive Xpert Ultra result were reclassified as tuberculosis-negative only if they had a history of tuberculosis (n=13).

‡ Study participants testing tuberculosis-positive based on a trace-positive Xpert Ultra result had Xpert Ultra testing on a subsequent sputum specimen: if the subsequent sputum Xpert Ultra result was negative for *M tuberculosis* then the participant was reclassified as tuberculosis-negative; if the subsequent Xpert Ultra result was positive for *M tuberculosis* (any semiquantitative threshold), then the participant was not reclassified and remained tuberculosis-positive (14 out of 32 participants tested tuberculosis-negative on sample 2 and were reclassified; 14 tested tuberculosis-positive on sample 2 and were not reclassified; and four were were non-determinate by Xpert Ultra on sample 2 and were not reclassified).

19 (44%) of 43 participants with a positive Xpert Ultra test but no positive culture had an Xpert Ultra semiquantitative readout of trace. 15 (35%) participants with apparent false-positive Xpert Ultra results were also positive by Xpert (appendix p 9). Two (5%) participants with apparent false-positive Xpert Ultra results had *M tuberculosis* identified on a follow-up culture, and two (5%) participants were treated for tuberculosis on the basis of clinical suspicion. Of the 24 (56%) participants who did not have culture-positive or Xpert-positive sputum and who had not started therapy, 18 participants gave 2-month follow-up information on symptoms. Symptoms had resolved in nine participants, improved in eight participants, and had not changed in one participant. Sequencing of the amplicons obtained from 14 cartridges (14 participants) with apparent false-positive results showed *M tuberculosis* DNA in 12 participants (appendix pp 10–12).

In a post-hoc analysis, we explored the effect of reclassifying Xpert Ultra trace-positive results as tuberculosis-negative on sensitivity and specificity for case detection (appendix p 7). Eliminating the trace-positive category and reclassifying all trace-positive results as tuberculosis-negative improved Xpert Ultra specificity but reduced its sensitivity (table 3). A conditional-trace approach (Xpert Ultra trace-positive results were reclassified as tuberculosis-negative only in participants with a history of tuberculosis) and a trace-repeat approach (participants with a trace-positive Xpert Ultra result for the first specimen were classified either as tuberculosis-negative if an Xpert Ultra test result of another sputum specimen was negative, or as tuberculosis-positive if an Xpert Ultra test result of another sputum specimen was positive) also improved Xpert Ultra specificity estimates (table 3). The conditional trace and trace-repeat approaches retained most of Xpert Ultra's sensitivity in the smear-negative group. In a post-hoc analysis stratified by country-specific tuberculosis incidence, the specificity of Xpert Ultra was almost identical to that of Xpert in countries with low incidence (100 cases per 100 000 population or less), whereas the difference between Xpert Ultra and Xpert was greatest (−8% [95% CI −14 to −5] in favour of Xpert) in participants with a medical history of tuberculosis who were enrolled in countries with high tuberculosis incidence (more than 100 cases per 100 000 population; appendix p 13).

On initial testing of 2001 specimens, non-determinate readouts (invalid, error, no result) were obtained for 39 (2%) specimens with Xpert and for 79 (4%) specimens with Xpert Ultra. After excluding instrument-related errors, non-determinate readouts were obtained for 28 (1%) specimens with Xpert and for 64 (3%) specimens with Xpert Ultra. A single repeat test done on the same specimen that initially was non-determinate reduced the number of non-determinate results to four (<1%) specimens with Xpert and to ten (<1%) specimens for Xpert Ultra.

## Discussion

Results of this multicentre diagnostic accuracy study show that the sensitivity of Xpert Ultra was superior to that of the standard Xpert for tuberculosis case detection in participants with sputum smear-negative pulmonary tuberculosis. Xpert Ultra also had superior sensitivity for tuberculosis case detection in HIV-infected participants and in all study participants. In clinical practice, the high sensitivity of Xpert Ultra could facilitate diagnosis of tuberculosis at earlier stages of disease and diagnosis of tuberculosis in patients with HIV and sputum smear-negative tuberculosis, a population with high mortality. Similarly, sensitivity gains could also be relevant for diagnosis of tuberculosis in children and for diagnosis of extrapulmonary forms of tuberculosis such as meningitis. These groups were assessed in separate studies.^19^, ^20^

The increased sensitivity of Xpert Ultra came at the expense of a loss of specificity. For Xpert Ultra, we observed a difference in specificity between patients with and without a medical history of tuberculosis treatment. Xpert Ultra specificity increased with increasing time since completion of treatment since the preceding tuberculosis episode up to 7 years. Xpert specificity differed by tuberculosis treatment history only if the preceding treatment had been completed within the past 2 years. These results are in line with findings by Theron and colleagues^21^ that show that Xpert-positive, culture-negative results were more common in individuals with a history of tuberculosis. Extraneous *M tuberculosis* from other specimens or the laboratory environment, or false-negative cultures from over-decontamination are possible explanations for a positive nucleic-acid amplification test result in participants with sputum cultures that are negative for *M tuberculosis*. However, in our study, over-decontamination is not sufficient to explain all of the specificity decrement for Xpert Ultra, and environmental contamination is an unlikely explanation because we implemented rigorous laboratory quality assurance and quality monitoring throughout the study. We speculate that in our study, most instances of Xpert Ultra-positive, culture-negative results were caused by the presence of *M tuberculosis* DNA or intact *M tuberculosis* bacilli (either living or dead, originating from the participant's lower respiratory system), or both in sputum. *M tuberculosis* mRNA has also been detected in sputum along with persisting PET thoracic lesion activity in some patients with tuberculosis 1 year after standard 6-month tuberculosis treatment.^22^ It remains to be seen whether apparent reductions in test specificity in patients with a history of tuberculosis will also be observed for other molecular tests for tuberculosis that aim to improve sensitivity through the detection of multicopy targets.^23^, ^24^ Additional studies with longer follow-up that investigate the natural history of patients with Xpert Ultra-positive and culture-negative results are needed to understand the clinical relevance of these test results.

More than half of Xpert Ultra false-positive results in patients with a history of tuberculosis were trace-positive (the semiquantitative result corresponding to the lowest bacillary burden), so reclassification of these results as tuberculosis-negative could be considered for all patients, for patients with a tuberculosis history only, or on the basis of Xpert Ultra test results from another sputum specimen. These approaches mitigate some loss of Xpert Ultra specificity while maintaining some sensitivity gains over Xpert. The population-level effect of the sensitivity and specificity trade-off on patient-important outcomes would be expected to vary by setting. For Xpert Ultra, country-level tuberculosis incidence levels seem to affect test specificity. For example, in our study, Xpert Ultra specificity was 99% in participants without a history of tuberculosis treatment in study sites in countries where the tuberculosis incidence of 100 cases per 100 000 population or less, and 95% in patients without tuberculosis treatment history in countries where the tuberculosis incidence is more than 100 cases per 100 000 population.^1^ Modelling studies are underway and will allow more in-depth exploration of the trade-offs between increased numbers of patients correctly and falsely diagnosed under different epidemiologic scenarios.

For detection of rifampicin resistance, Xpert Ultra specificity was non-inferior to that of Xpert. The sensitivity point estimate for Xpert Ultra was slightly less than that of Xpert and the confidence interval was wide, such that non-inferiority criteria were not met. Additional studies including larger numbers of rifampicin-resistant specimens are needed to more precisely characterise Xpert Ultra accuracy for detection of rifampicin resistance. Patients belonging to the multidrug-resistance risk group were recruited mainly from four sites and, accordingly, most rifampicin-resistant cases come from these sites. Mutations such as Ile491Phe, which is not detected by Xpert or Xpert Ultra, might be more common in countries not included in this study, and inclusion of such sites could potentially have reduced sensitivity estimates. However, we found no evidence of bias given that our reported accuracy for rifampicin-resistance detection is equivalent to that reported in a Cochrane review^4^ of a broad group of studies and sites worldwide. Our estimates are also in line with WHO surveillance data on the frequency of rifampicin-resistance-conferring mutations obtained from resistance surveys.

In summary, Xpert Ultra holds promise as a rapid and highly sensitive test for tuberculosis case detection and simultaneous detection of rifampicin resistance. Its sensitivity gain compared with Xpert is most apparent in individuals with low sputum bacillary burdens. Implementation approaches will need to consider the effect of possible false-positive Xpert Ultra results.

**This online publication has been corrected. The corrected version first appeared at thelancet.com/infection on February 21, 2018.**
