# Supplementary material for: Xpert MTB/RIF Ultra for detection of Mycobacterium tuberculosis and rifampicin resistance: a prospective multicentre diagnostic accuracy study
Source: Lancet Infect Dis. 2018 Jan;18(1):76–84. doi: 10.1016/S1473-3099(17)30691-6 (PMC6168783; doi:10.1016/S1473-3099(17)30691-6)
Supplement: Supplementary appendix [file mmc1.pdf]

THE LANCET
Infectious Diseases

Supplementary appendix

This appendix formed part of the original submission and has been peer reviewed. We post it as supplied by the authors.

Supplement to: Dorman S E, Schumacher S G, Alland D, et al. Xpert MTB/RIF Ultra for detection of *Mycobacterium tuberculosis* and rifampicin resistance: a prospective multicentre diagnostic accuracy study. *Lancet Infect Dis* 2017; published online Nov 30. http://dx.doi.org/10.1016/S1473-3099(17)30691-6.

Supplementary webappendix

Supplement to: Dorman SE, Schumacher SG, Alland DA, et al. Xpert MTB/RIF Ultra for detection of *Mycobacterium tuberculosis* and rifampin resistance: a prospective multicentre diagnostic accuracy study

Table of Contents

Supplementary text

Sequencing of IS6110, IS1081, and rpoB from Ultra cartridge amplicons.

Criteria for site selection

Sample size calculations

Supplementary figure

S1. Graphical representation of sensitivity and specificity differences between Ultra and Xpert, with 95% confidence intervals

Supplementary tables

S1. Sensitivity of a single liquid culture compared to Xpert and Ultra.

S2. Sensitivity of Ultra and Xpert, using different case definitions.

S3. Test sensitivity and specificity depending on tuberculosis history and different approaches to interpretation of Ultra trace-positive semiquantitative results for *M. tuberculosis* detection

S4. Ultra specificity for tuberculosis case detection, stratified by personal tuberculosis history

S5. Details for 43 participants with Ultra result of "MTB detected" on initial sputum, but no culture positive for M. tuberculosis.

S6. Ultra specificity for tuberculosis case detection in patients without history of prior tuberculosis, by country incidence of tuberculosis

S7. Rifampin drug susceptibility test results for Ultra and Xpert, for 684 participants with phenotypic drug susceptibility test results.

S8. Details for participants with discordant rifampin drug susceptibility test results by Ultra and phenotypic drug susceptibility testing.

S9. Cross-tabulation of results of Xpert and Ultra.

S10. Cross-tabulation of semiquantitative results of Xpert and Ultra.

S11. Xpert and Ultra positive predictive value for tuberculosis case detection, stratified by personal tuberculosis history

Supplementary documents

Study Protocol (to be published on the authors' institutional website)

Statistical Analysis Plan (to be published on the authors' institutional website)

Supplementary text

Sequencing of IS6110, IS1081, and rpoB from Ultra cartridge amplicons.

All FIND-coordinated sites were instructed to store positive Ultra cartridges for further assessment of discrepant cases. Available samples from discordant cases for tuberculosis detection and the same number of randomly selected non-discordant cases were shipped either to Italy (Ospedale San Raffaele, Milan) or India (PD Hinduja Hospital, Mumbai). Residual DNA lysate was retrieved directly from each positive Ultra cartridge following a procedure that involved the transfer of residual buffer from one cartridge chamber to another and running the Ultra cartridge with a specific assay definition file. The procedure was conducted in a biosafety cabinet in a separate area and under controlled conditions for high risk of DNA and amplicon contamination. The amplicons were then used for testing by next generation sequencing (Illumina Miniseq System, analysis by PhyResSe [Ref 1]) and pyrosequencing (Qiagen PyroMark Q96 ID, analysis by IdentiFire [Ref 2]) in Italy and India, respectively.

References

1. Feuerriegel S, Schleusener V, Beckert P, Kohl TA, Miotto P, Cirillo DM, et al. PhyResSE: a Web Tool Delineating Mycobacterium tuberculosis Antibiotic Resistance and Lineage from Whole-Genome Sequencing Data. Carroll KC, editor. J Clin Microbiol. American Society for Microbiology; 2015;53: 1908–1914. doi:10.1128/JCM.00025-15

2. Ajbani K, Lin S-YG, Rodrigues C, Nguyen D, Arroyo F, Kaping J, et al. Evaluation of pyrosequencing for detecting extensively drug-resistant Mycobacterium tuberculosis among clinical isolates from four high-burden countries. Antimicrobial Agents and Chemotherapy. American Society for Microbiology; 2015;59: 414–420. doi:10.1128/AAC.03614-14

Criteria for site selection

Study sites were selected to ensure representativeness of the global TB epidemic. As such the selection of study sites balanced several factors including a) ensuring that the study population was representative of the target population for the Ultra test (e.g. high TB burden setting including persons living with HIV); b) representation of settings with high drug resistance; and c) capacity of sites to undertake the rigorous reference standard laboratory work. Participants from diverse high burden populations were recruited in countries including settings with: i) high notification rates of tuberculosis in referral centers (all sites, in addition to primary care tuberculosis clinics in Cape Town and Kisumu) ii) high rates of multidrug resistance in tertiary care and drug resistance referral centers (Minsk, Zhengzhou, Tbilisi, Mumbai and New Delhi); and iii) high incidence of HIV infection (all African sites).

Sample size calculations.

We chose the margin for sensitivity in smear-negative TB based on the following general principles and basic rationale. Given the existing uncertainty, we aimed to choose a trial size such that:

- For non-inferiority to be demonstrated, the point estimate should still suggest superiority (i.e. point estimate ≥0%, i.e. increased sensitivity)
- If our assumptions were to be met exactly, superiority would be shown
- When simulating a large number of trials based on our assumptions, ≥80% of simulated trials would lead to a conclusion of non-inferiority based on the chosen NI-margin

Generic sample size formulas do not account for the correlation between tests that is present when testing samples from the same patient with two tests. Additionally, such formulas rely on asymptotic theory that yields biased results for small sample sizes. We therefore carried out sample size calculations via Monte-Carlo Simulation. For all simulations, we conservatively assumed a moderate correlation of 0.5 between the tests. Using the parameter values specified in the table we generated 10,000 correlated binary data sets for each simulation. Our criterion for choosing the final size was that the desired study outcome of non-inferiority was shown in at least 80% of simulated data sets. Once a sample size fulfilled this criterion, at least two additional simulations were run using the same parameter inputs to verify the stability of the simulation result. If results were unstable between repeated simulations, the process was repeated with an increased number of simulated data sets (e.g. 50,000) per simulation. The same was done if the simulation results did not calibrate well with input parameters of if the histograms of output parameters did not have a smooth distribution.

Sample size calculations for test sensitivity for tuberculosis case detection in participants with *M. tuberculosis* cultured from sputum but no sputum smear positive for acid fast bacilli (sm neg/cx pos), assuming that Xpert and Ultra have different test sensitivities, and non-inferiority margin is 7%					
Sensitivity of Xpert in sm neg/cx pos	Sensitivity of Ultra in sm neg/cx pos	% difference	# of sm neg/cx pos TB cases needed	Total # of cx pos TB cases needed assuming 30% sm neg/cx pos	Total # of enrolled participants assuming TB prevalence 20% among enrollees
65	75	10	60	200	1000
70	80	10	55	183	917
75	85	10	48	160	800
80	90	10	39	130	650

Sample size calculations for test sensitivity for detection of rifampin resistance, assuming that Xpert and Ultra have the same sensitivity, and non-inferiority margin is 3%				
Sensitivity of Xpert for detection of rifampin resistance	Sensitivity of Ultra for detection of rifampin resistance		# of rifampin resistant TB cases needed	% of participants with rifampin resistance
90	90	0	618	25%
95	95	0	327	12%
98	98	0	135	5%

Sample size calculations for test specificity for detection of rifampin resistance, assuming that Xpert and Ultra have the same specificity, and non-inferiority margin is 3%				
Specificity of Xpert for detection of rifampin resistance	Specificity of Ultra for detection of rifampin resistance		# of rifampin susceptible TB cases needed	% of participants with rifampin resistance
97	97	0	450	16%
98	98	0	303	11%
99	99	0	154	6%

Figure S1. Graphical representation of sensitivity and specificity differences between Ultra and Xpert, with 95% confidence intervals

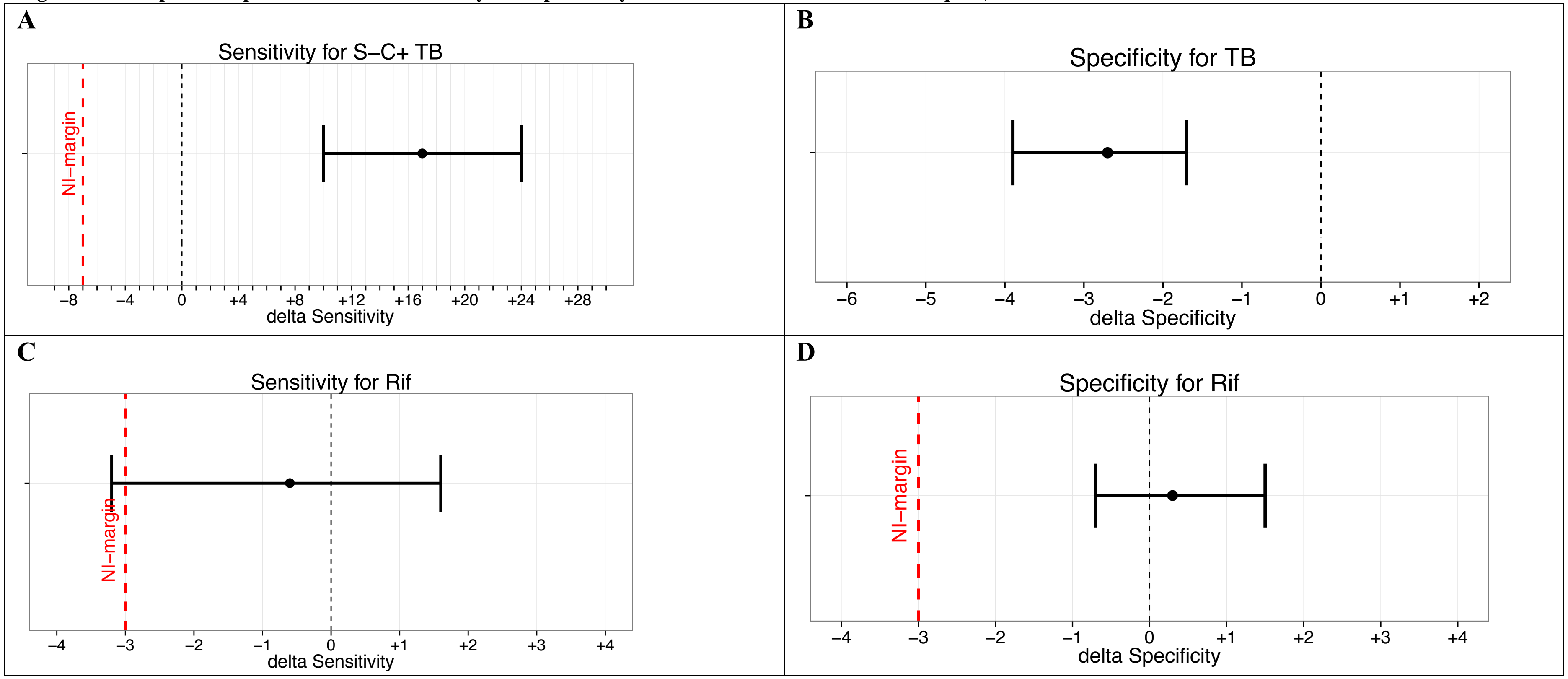

The difference in sensitivity/specificity (Δ = Ultra – Xpert) is displayed as horizontal lines with the point representing the point estimate and whiskers representing the upper and lower limit of the 95%CIs of Δ. The black vertical dotted line indicates zero difference in sensitivity/specificity and the red vertical broken line indicates the non-inferiority margin. **Panel A** shows the difference in sensitivity for the detection of smear-negative/culture-positive tuberculosis. The lower limit of the 95%CI (+10%) lies above the non-inferiority margin of -7%, demonstrating non-inferiority of Ultra to Xpert; the lower limit of the 95%CI also lies above 0% (the point of no difference), thus also showing superiority of Ultra sensitivity over Xpert. **Panel B** shows the difference in specificity for the detection of tuberculosis. A non-inferiority margin had not been pre-specified for this comparison, so an assessment of non-inferiority could not be done (and no non-inferiority margin is shown). However, the upper limit of the 95%CI lies below 0% (the point of no difference), suggesting that specificity of Ultra was inferior to that of Xpert. **Panel C** shows the difference in sensitivity for the detection of rifampin-resistance. The lower limit of the 95%CI (-3·6%) lies below the non-inferiority margin of -3%, thus non-inferiority of Ultra to Xpert could not be demonstrated. **Panel D** Shows the difference in specificity for the detection of rifampin-resistance. The lower limit of the 95%CI (-0·9%%) lies above the non-inferiority margin of -3%, demonstrating non-inferiority of Ultra to Xpert.

Table S1. Sensitivity of a single liquid culture compared to Xpert and Ultra.

	Analysis 1[1]		Analysis 2[2]		Analysis 3[3]		Analysis 4[4]		Analysis 5[5]	
	**All culture-positive**	**Smear-negative, culture-positive**	**All culture-positive** (n=443)	**Smear-negative, culture-positive** (n=131)	**All culture-positive** (n=384)	**Smear-negative, culture-positive** (n=119)	**All culture-positive** (n=370)	**Smear-negative, culture-positive** (n=108)	**All culture-positive** (n=363)	**Smear-negative, culture-positive** (n=98)
**Xpert on S1**	83·1% (399/480)	46·1% (65/141)	83·5%	46·6%	82·8%	47·1%	84·1%	48·1%	87·1%	55·1%
**Ultra on S1**	88·0% (417/474)	62·0% (88/142)	88·3%	62·8%	87·2%	59·7%	88·4%	61·1%	91·2%	68·4%
**MGIT on S2**	91·7% (433/472)	73·4% (102/139)	91·6%	72·5%	90·9%	71·4%	90·5%	68·5%	NA	NA
**MGIT on S3**	92·3% (395/428)	78·8% (104/132)	NA	NA	92·4%	79·0%	NA	NA	92·0%	74·5%
**Ultra - Xpert**	+4·9%	+15·9%	+4·8%	+16·2%	+4·4%	+12·6%	+4·3%	+13·0%	+4·1%	+13·3%
**Ultra - MGIT on S2**	-3·7%	-11·4%	-3·3%	-9·7%	-3·7%	-11·7%	-2·1%	-7·4%	NA	NA
**Ultra - MGIT on S3**	-4·3%	-16·8	NA	NA	-5·2%	-19·3%	NA	NA	-0·8%	-6·1%

Note: Sensitivity of Xpert on S1 and Ultra on S1 as per primary analysis were 82·9% and 88·3%, respectively, in all culture-positive participants (n=462) and 46·0% and 62·8%, respectively, in smear-negative, culture-positive participants (n=137)

[1] Includes all participants with valid results on each individual test, i.e. analysis not limited to participants with valid results on all four tests (thus denominators vary by assay and are shown in each cell)

[2] Includes only participants with valid results on Xpert on S1, Ultra on S1 and MGIT on S2; participants with missing/contaminated results for MGIT on S3 were not excluded from the analysis (thus results from MGIT on S3 were not available for all of the 443/131 participants in this group and sensitivity of MGIT on S3 is not estimated)

[3] Includes participants with valid results on Xpert on S1, Ultra on S1, MGIT on S2 and MGIT on S3

[4] Includes participants with valid results on all tests (Xpert on S1, Ultra on S1, MGIT on S2 and MGIT on S3) and excluding MGIT on S2 from the reference standard to avoid incorporation bias; sensitivity of MGIT on S3 is not estimated in this group because no liquid culture results would be available as part of the reference standard, which would lead to biased/misleading estimates.

[5] Includes all patients with valid results on all tests (Xpert on S1, Ultra on S1, MGIT on S2 and MGIT on S3) and excluding MGIT S3 from the reference standard; sensitivity of MGIT on S2 is not estimated in this group because no liquid culture results would be available as part of the reference standard, which would lead to biased/misleading estimates.

Table S2. Sensitivity of Ultra and Xpert, using different tuberculosis case definitions.

	Definition of reference standard					
	2 MGIT cultures + 2 LJ cultures + cultures from sputum #4 and month 2 follow-up	2 MGIT cultures + 2 LJ cultures	1 MGIT culture + 1 LJ culture	1 MGIT culture		
Xpert	45%	46%*	60%	61%	3 smears[1]	Definition of smear-result
	50%	51%	66%	67%	3 smears[2]	
	58%	59%	73%	74%	1 smear[3]	
Ultra	63%	63%*	76%	76%	3 smears[1]	
	67%	67%	79%	79%	3 smears[2]	
	72%	72%	84%	84%	1 smear[3]	

*estimates from the current study.

[1] A participant was considered sputum smear microscopy positive if at least one of 3 smears had semi-quantitative grade of scanty or higher.
[2] A participant was considered sputum smear microscopy positive if at least one of 3 smears had semi-quantitative grade of 1+ or higher, or at least 2 smears had semi-quantitative grade of scanty or higher (definition used in Boehme et al NEJM, 2010)
[3] A participant was considered sputum smear microscopy positive if the same sputum as used for Ultra and Xpert was smear-positive at a semi-quantitative grade of scanty or higher.

Notes: Xpert and Ultra results are from S1, single MGIT result and results from 1 MGIT + 1 LJ are from S2; cultures from sputum #4 are the MGIT and LJ cultures that were done on the fourth sputum specimen if Xpert and Ultra were discordant; "month 2 follow-up cultures" are the MGIT and LJ study cultures that were done at the 2-month follow-up visit for patients that had not started TB treatment (obtaining sputum for 2-month follow-up cultures was attempted for all such patients in Brazil, Cape Town, China, Kenya and Uganda; for the remaining sites this was attempted for 10% of such patients who were negative on culture and Xpert). Xpert specificity was 98% for all definitions; Ultra specificity was 96% when using two MGITs and two LJs or more and 95% when using only one MGIT and one LJ culture or less.

Abbreviations: MGIT, Mycobacterial growth indicator tube; LJ, Lowenstein-Jensen

Table S3. Test sensitivity and specificity depending on tuberculosis history and different approaches to interpretation of Ultra trace-positive semiquantitative results for *M. tuberculosis* detection

Comparison	Difference in Sensitivity (95%CI)		Difference in Specificity (95%CI)		
	All culture-positive (n=462)	Smear-negative, culture-positive (n=137)	All culture-negative (n=977)	No history of tuberculosis (n=727)	Any history of tuberculosis (n=249)
**Ultra -Xpert (base case)**	**+5·4%** (+3·3, +8·0) [25/462]	**+17%** (+10, +24) [23/137]	**-2·7%** (-3·9, -1·7) [26/977]	**-1·9%** (-3·3, -0·9) [14/727]	**-4·8%** (-8·2, -2·8) [12/249]
**Ultra 'no trace'** [1] **- Xpert**	**+2·6%** (+0·6, +4·9) [12/462]	**+8·0%** (+1·6, +15) [11/137]	**-0·7%** (-1·6, -0·01) [7/977]	**-0·8%** (-1·9, +0·03) [6/727]	**-0·4%** (-2·5, +1·5) [1/249]
**Ultra 'conditional trace'** [2] **- Xpert**	**+5·0%** (+2·9, +7·5) [23/462]	**+15%** (+9·1, +23) [21/137]	**-1·5%** (-2·6, -0·7) [15/977]	**-1·9%** (-3·3, -0·9) [14/727]	**-0·4%** (-2·5, +1·5) [1/249]
**Ultra 'trace-repeat'** [3] **- Xpert**	**+4·5%** (+2·6, +7·0) [21/462]	**+15%** (+8·4, +22) [21/137]	**-1·6%** (-2·7, -0·8) [16/977]	**-1·1%** (-2·2, -0·2) [8/727]	**-3·2%** (-6·4, -0·9) [8/249]

Note: Round brackets show 95%CIs and square brackets show numerators/denominators. Sensitivity varied little by TB history and not systematically. Specificity did not vary between smear-negative and smear-positive study participants. Data on TB history was unavailable for one patient.

[1] Study participants testing tuberculosis-positive based on a trace-positive Ultra result (n=32) were reclassified as tuberculosis-negative

[2] Study participants testing tuberculosis-positive based on a trace-positive Ultra result were reclassified as tuberculosis-negative only if they had a history of tuberculosis (n=13)

[3] Study participants testing tuberculosis-positive based on a trace-positive Ultra result had Ultra testing performed on a subsequent sputum specimen: if the subsequent sputum Ultra result was MTB-negative then the participant was reclassified as tuberculosis-negative; if the subsequent Ultra result was MTB-positive (any semi-quantitative threshold), then the participant was not reclassified and remained tuberculosis-positive (14 out of 32 participants tested tuberculosis-negative on sample 2 and were reclassified; 14 and tested tuberculosis-positive on sample 2 and were not reclassified; and 4 out of 32 were Ultra non-determinate on sample 2 and were not reclassified)

Table S4. Xpert and Ultra specificity (95%CI) for tuberculosis case detection, stratified by personal tuberculosis history

	Time since prior tuberculosis episode			No prior history	Any prior history
	**≤2 years (n=55)**	**>2 years & ≤5 years (n=63)**	**>5 years (n=108)**	**(n=727)**	**(n=249)**
**Xpert**	**92·7%** (82·4, 98·0)	**100%** (94·3, 100)	**99·1%** (94·9, 100)	**98·3%** (97·1, 99·1)	**98·0%** (95·4, 99·3)
**Ultra**	**83·6%** (71·2, 92·2)	**93·7%** (84·5, 98·2)	**96·3%** (90·8, 99·0)	**96·4%** (94·8, 97·7)	**93·2%** (89·3, 96·0)
**Difference** (Ultra – Xpert)	**-9·1%**	**-6·3%**	**-2·8%**	**-1·9%**	**-4·8%**

Note: Time since prior history was unknown for 23 individuals with negative cultures.

Table S5. Details for 43 participants with Ultra MTB detected result on initial sputum, but no positive culture result on the specimens used for the reference standard.

Participant ID	Site	Ultra semi-quantitative result[1]	Xpert result	HIV status	Prior history TB (months)[2]	CXR result	MTB DNA sequencing[3]	TB treatment initiated[4]	Signs/ symptoms at follow-up[5]	Culture result at follow-up[6]
730058	Belarus	Trace	Negative	ND	No	TB likely	IS6110-pos IS1081-pos	LTFU	NA	Positive for MTB[10]
007ZN0003	Cape Town	Trace	Negative	Positive	Yes (16)	ND	ND	NA	Completely recovered	ND
007ZN0097	Cape Town	Very low	Very low	Negative	Yes (11)	ND	ND	Yes	Improved	Negative
007ZN0116	Cape Town	Low	Low	Negative	Yes (12)	ND	ND	LTFU	NA	ND
007ZN0161	Cape Town	Very low	Negative	Negative	Yes (56)	ND	ND	NA	Completely recovered	Negative
007ZN0162	Cape Town	Trace	Negative	Negative	Yes (24)	ND	ND	NA	Completely recovered	Negative
007ZN0168	Cape Town	Very low	Negative	Positive	Yes (144)[7]	ND	ND	NA	Improved	Negative
670037	Georgia	Low	Negative	ND	No	Pneumonia (TB unlikely)	IS6110-neg IS1081-neg[8]	No	Completely recovered	ND
670126	Georgia	Trace	Very low	ND	Yes (143)	TB likely	IS6110-pos IS1081-pos	Yes	Completely recovered	ND
670131	Georgia	Trace	Negative	ND	No	TB likely	IS6110-pos	Yes	Unchanged	ND
670205	Georgia	Low	Low	ND	Yes (12)	TB likely	IS6110-pos IS1081-pos rpoB-pos	Yes	Improved	ND

Participant ID	Site	Ultra semi-quantitative result[1]	Xpert result	HIV status	Prior history TB (months)[2]	CXR result	MTB DNA sequencing[3]	TB treatment initiated[4]	Signs/ symptoms at follow-up[5]	Culture result at follow-up[6]
670235	Georgia	Very low	Very low	ND	Yes (19)	Bronchitis (TB unlikely)	IS6110-pos IS1081-pos rpoB-pos	No[11]	Completely recovered	ND
651017	Johannesburg	Trace	Negative	Positive	No	ND	IS6110-pos IS1081-pos	No	Completely recovered	Negative
652022	Johannesburg	Trace	Negative	Positive	Yes (29)	ND	IS6110-pos IS1081-pos	LTFU	NA	ND
007KE0002	Kenya	Very low	Very low	Positive	No	ND	ND	LTFU	NA	ND
007KE0006	Kenya	Very low	Negative	Negative	No	ND	ND	NA	Improved	Negative
007KE0086	Kenya	Trace	Negative	Positive	Yes (78)	TB likely	ND	NA	Improved	Negative
007KE0093	Kenya	Trace	Negative	Positive	Yes (6)	ND	ND	NA	Unchanged	ND
007KE0121	Kenya	Low	Very low	Positive	No	ND	ND	LTFU	NA	ND
007KE0130	Kenya	Trace	Negative	Negative	No	ND	ND	NA	Improved	Negative
007KE0174	Kenya	Very low	Negative	Positive	No	ND	ND	NA	Improved	Negative
420138	Mumbai	Trace	Negative	ND	No	TB likely	IS6110-neg[9]	LTFU	NA	ND
420194	Mumbai	Very low	Very low	ND	No	ND	IS6110-pos	LTFU	NA	ND
490009	New Delhi	Very low	Negative	ND	No	ND	IS6110-pos	No	Completely recovered	Negative
490032	New Delhi	Trace	Negative	ND	Yes (62)	ND	IS6110-pos	LTFU	NA	Negative
490064	New Delhi	Very low	Very low	ND	No	ND	IS6110-pos	LTFU	NA	ND
490067	New Delhi	Very low	Very low	Negative	No	ND	ND	LTFU	NA	ND

Participant ID	Site	Ultra semi-quantitative result[1]	Xpert result	HIV status	Prior history TB (months)[2]	CXR result	MTB DNA sequencing[3]	TB treatment initiated[4]	Signs/ symptoms at follow-up[5]	Culture result at follow-up[6]
490082	New Delhi	Trace	Negative	Positive	Yes (125)	TB likely	IS6110-pos	LTFU	NA	ND
490100	New Delhi	Trace	Negative	ND	Yes (13)	TB likely	IS6110-pos	LTFU	NA	Negative
490101	New Delhi	Very low	Very low	Negative	No	ND	IS6110-pos	LTFU	NA	ND
490110	New Delhi	Very low	Very low	Negative	No	ND	IS6110-pos	LTFU	NA	ND
490119	New Delhi	Very low	Negative	Negative	No	TB likely	NI[β]	LTFU	NA	ND
007UK2076	Uganda	Very low	Very low	Negative	No	ND	ND	LTFU	NA	ND
007UK2086	Uganda	Trace	Negative	Positive	No	ND	ND	NA	NA	ND
007UK2107	Uganda	Very low	Very low	Negative	No	ND	ND	Yes	Improved	ND
007UK2128	Uganda	Very low	Very low	Negative	No	ND	ND	LTFU	NA	ND
007UK2132	Uganda	Trace	Negative	Negative	No	ND	ND	NA	Improved	Negative
007UK2135	Uganda	Very low	Negative	Positive	No	ND	ND	NA	Completely recovered	Negative
007UK2142	Uganda	Trace	Negative	Positive	Yes (35)	ND	ND	NA	Improved	Positive for MTB
007UK2143	Uganda	Trace	Negative	Negative	Yes (35)	ND	ND	NA	Improved	ND
007UK2145	Uganda	Trace	Negative	Negative	No	ND	ND	NA	Improved	Negative
007UK2151	Uganda	Very low	Negative	Positive	No	ND	ND	Yes	Improved	Negative
007UK2158	Uganda	Very low	Negative	Negative	No	ND	ND	NA	NA	ND

NA=Not available. ND=Not done or only contaminated cultures. NI=results not interpretable (in cases where the quality of DNA was poor or due to the presence of inhibitors the sequencing results showed a score of less than 95%; as per laboratory protocol any score below 95% was considered NI (the ideal score being 100 for a valid result); all for specimens with initial NI results the test was repeated twice before reporting the result as NI. LTFU=lost to follow-up.

[1]Ultra semi-quantitative results from sputum 1

[2]History of prior tuberculosis treatment (months since completion of prior tuberculosis treatment)

[3]Sequencing of Ultra cartridge amplicons for MTB done only at FIND sites by Next Generation Sequencing (NGS) in Belarus, Georgia and Johannesburg or by pyrosequencing in Hinduja and New Delhi. This identified results compatible with MTB in 14 out of 16 valid sequencing results.

[4]Anti-tuberculosis treatment initiated, based on information obtained approximately 2 months after enrolment (information available only for a subset of patients, refer to study protocol for additional details)

[5]Signs/symptoms compared to baseline, approximately 2 months after enrollment (information available only for a subset of patients, refer to study protocol for additional details)

[6]Results of culture of sputum obtained at approximately 2 months after enrollment (information available only for a subset of patients, refer to study protocol for additional details)

[7]Actual treatment end date unknown

[8]Sequencing of Ultra cartridge amplicons for MTB was done by NGS and sequences of IS6110 or IS1081 were not detected. The false-positive Ultra call resulted from a very late cycle threshold (CT) value on the probe detecting IS6110/1081.

[9]Sequencing of Ultra cartridge amplicons for MTB was done by pyrosequencing and sequences of IS6110 were not detected. The false-positive Ultra call resulted from a clear signal from IS6110/1081. However, since (i) primers aiming for IS1081 were not used in the pyrosequencing reaction, and (ii) strains not containing IS6110 but containing IS1081 are not uncommon in India, this result does not provide conclusive evidence of the absence of MTB DNA in the original patient specimen.

[10]Culture-positive for *M. tuberculosis* from sputum obtained during a non-study clinical assessment

[11]Xpert-negative from sputum obtained during routine assessment, continued to be asymptomatic 6 months after enrolment

Table S6. Ultra specificity for tuberculosis case detection, stratified by country incidence of tuberculosis and personal tuberculosis history status

	Specificity for tuberculosis case detection in patients without a history of prior tuberculosis		Specificity for tuberculosis case detection in patients with a history of prior tuberculosis	
**Country annual tuberculosis incidence**	**≤100/100,000 population[1]**	**>100/100,000 population[2]**	**≤100/100,000 population[1]**	**>100/100,000 population[2]**
**Xpert**	**99·6%** (95%CI 98·0, 100)	**97·6%** (95%CI 95·7, 98·8)	**97·0%** (95%CI 91·6, 99·4)	**98·6%** (95%CI 95·2, 99·8)
**Ultra**	**98·9%** (95%CI 96·9, 99·8)	**94·9%** (95%CI 92·4, 96·7)	**97·0%** (95%CI 91·6, 99·4)	**90·5%** (95%CI 84·6, 94·7)
**Difference** (Ultra – Xpert)	**-0·7%** (95%CI -2·8, +1·0)	**-2·7%** (95%CI -4·7, -1·2)	**0·0%** (95%CI -3·7, +3·7)	**-8·1%** (95%CI -13·6, -4·7)

[1]study sites (TB incidence per 100,000 population): Brazil (41/100k), Belarus (55/100k), China (67/100k), Georgia (99/100k)
[2]study sites (TB incidence per 100,000 population): Uganda (202/100k), India (217/100k), Kenya (233/100k), and South Africa (834/100k)

Table S7. Rifampin drug susceptibility test results for Ultra and Xpert, for 684 participants with phenotypic drug susceptibility test results

		Rifampin phenotypic DST result		
		**Susceptible (n=471)**	**Resistant (n=213)**	**Total (n=684)**
**Ultra**				
	**Rifampin resistance detected**	1·5% (7/471)	81% (172/213)	**179/684**
	**Rifampin resistance not detected**	84% (397/471)	5·6% (12/213)	**409/684**
	**Rifampin resistance indeterminate**	2·6% (12/471)	1·9% (4/213)	**16/684**
	**MTB not detected**	7·9% (37/471)	8·9% (19/213)	**56/684**
	**MTB non-determinate***	3·8% (18/471)	2·8% (6/213)	**24/684**
**Xpert**				
	**Rifampin resistance detected**	1·5% (7/471)	82% (174/213)	**181/684**
	**Rifampin resistance not detected**	83% (391/471)	3·8% (8/213)	**399/684**
	**Rifampin resistance indeterminate**	0·6% (3/471)	0·5% (1/213)	**4/684**
	**MTB not detected**	12% (58/471)	13% (27/213)	**85/684**
	**MTB non-determinate***	2·5% (12/471)	1·4% (3/213)	**15/684**

*Result for MTB detection was invalid, error or no result, and therefore no result was reported for rifampin susceptibility/resistance determination

Table S8. Details for participants with discordant rifampin drug susceptibility testing results by Ultra and phenotypic testing

Participant ID	Site	Rifampin phenotypic DST result (1·0 µg/mL)	Xpert rifampin result (Sputum 1)	Ultra rifampin result (Sputum 1)	DNA sequencing method	Mutation[1]	Confidence[2]
730116	Belarus	Susceptible	Resistance detected	Resistance detected	NGS	CAC526AAC	Minimal
CH0022	China	Susceptible	Resistance detected	Resistance detected	Sanger	CTG511CCG	Minimal
CH0080	China	Susceptible	Resistance detected	Resistance detected	Sanger	CTG533CCG	Moderate
CH0121	China	Susceptible	Resistance detected	Resistance detected	Sanger	CTG511CCG	Minimal
670050	Georgia	Susceptible	Resistance detected	Resistance detected	NGS	CTG533CCG	Moderate
652051	Johannesburg	Susceptible	NA	Resistance detected	NGS	CAC526TGC	Moderate
490007	New Delhi	Susceptible	Resistance detected	Resistance detected	Pyro	CAC526AAC	Minimal
ZN0156	Cape Town	Resistant	Resistance NOT detected	Resistance NOT detected	Sanger	Wild-type	-
CH0062	China	Resistant	Resistance NOT detected	Resistance NOT detected	Sanger	TCG531TTG	High
CH0106	China	Resistant	Resistance NOT detected	Resistance NOT detected	Sanger	TCG531TTG	High
CH0114	China	Resistant	Resistance NOT detected	Resistance NOT detected	Sanger	Wild-type	-
CH0119	China	Resistant	Resistance detected	Resistance NOT detected	Sanger	CAA513CCA	High
CH0123	China	Resistant	NA	Resistance NOT detected	Sanger	TCG531TTG	High
670384	Georgia	Resistant	NA	Resistance NOT detected	NGS	Wild-type	
490008	New Delhi	Resistant	Resistance NOT detected	Resistance NOT detected	Pyro	Wild-type	-
490017	New Delhi	Resistant	Resistance NOT detected	Resistance NOT detected	Pyro	Wild-type	-
490041	New Delhi	Resistant	Resistance NOT detected	Resistance NOT detected	Pyro	Wild-type	-
490108	New Delhi	Resistant	Resistance NOT detected	Resistance NOT detected	Pyro	Wild-type	-
490125	New Delhi	Resistant	NA	Resistance NOT detected	Pyro	indeterminate	

[1]mutation by DNA sequencing (*E. coli* numbering convention)
[2]mutation confidence of association with resistance
Abbreviations: DST, drug susceptibility testing; NGS, next-generation DNA sequencing methods; Pyro, pyrosequencing; NA, not available

Table S9. Cross-tabulation of results of Xpert and Ultra for MTB detection.

Cross-tabulation among culture-positive patients (including results indeterminate after repeat-testing). Numbers in cells represent numbers of participants.

		Xpert		
		**Indeterminate**	**Negative**	**Positive**
**Ultra**	**Indeterminate**	0	0	2
	**Negative**	1	54	3
	**Positive**	1	29	401

Cross-tabulation among culture-positive patients (as in primary analyses)

		Xpert	
		**Negative**	**Positive**
**Ultra**	**Negative**	51	3
	**Positive**	28	380

Cross-tabulation among culture-negative patients (including results indeterminate after repeat-testing). Numbers in cells represent numbers of participants.

		Xpert		
		**Indeterminate**	**Negative**	**Positive**
**Ultra**	**Indeterminate**	0	4	0
	**Negative**	1	979	2
	**Positive**	0	29	16

Cross-tabulation among culture-negative patients (as in primary analyses)

		Xpert	
		**Negative**	**Positive**
**Ultra**	**Negative**	932	2
	**Positive**	28	15

Table S10. Cross-tabulation of semiquantitative results of Xpert and Ultra.

Cross-tabulation of semiquantitative results of Xpert and Ultra: culture-positive patients. Numbers in cells represent numbers of participants.

		Xpert				
		**Negative**	**Very low**	**Low**	**Medium**	**High**
**Ultra**	**Negative**	51	2	1	0	0
	**Trace**	10	2	1	0	0
	**Very low**	12	20	4	0	0
	**Low**	6	13	19	5	6
	**Medium**	0	1	53	118	62
	**High**	0	0	1	31	44

Cross-tabulation of semiquantitative results of Xpert and Ultra: culture-negative patients. Numbers in cells represent numbers of participants.

		Xpert				
		**Negative**	**Very low**	**Low**	**Medium**	**High**
**Ultra**	**Negative**	932	2	0	0	0
	**Trace**	18	1	0	0	0
	**Very low**	9	11	0	0	0
	**Low**	1	1	2	0	0
	**Medium**	0	0	0	0	0
	**High**	0	0	0	0	0

Table S11. Xpert and Ultra positive predictive value (95%CI) for tuberculosis case detection, stratified by personal tuberculosis history

	Time since prior tuberculosis episode			No prior history	Any prior history
	≤2 years (n=52)	>2 years & ≤5 years (n=63)	>5 years (n=108)	(n=727)	(n=249)
**Xpert**	**76·5%** (50·1, 93·2)	**100%** (47·8, 100)	**90·9%** (58·7, 99·8)	**96·7%** (94·2, 98·3)	**86·8%** (71·9, 95·6)
**Ultra**	**66·7%** (44·7, 84·4)	**66·7%** (34·9, 90·1)	**71·4%** (41·9, 91·6)	**93·4%** (90·5, 95·6)	**69·6%** (55·9, 81·2)

Note: Time since prior history was unknown for 23 individuals with negative cultures.
